# Suspected cancer diagnoses made by general practitioners in a population with subsequently confirmed cancer diagnoses in Germany: a retrospective study of 31,628 patients

**DOI:** 10.18632/oncotarget.20886

**Published:** 2017-09-14

**Authors:** Karel Kostev, Uwe Meister, Matthias Kalder, Louis Jacob

**Affiliations:** ^1^ Epidemiology, QuintilesIMS, Frankfurt, Germany; ^2^ GP Praxis Meister, Nidderau, Germany; ^3^ Department of Gynecology and Obstetrics, Philipps-University Marburg, Marburg, Germany; ^4^ Faculty of Medicine, University of Paris 5, Paris, France

**Keywords:** cancer, suspected diagnosis, general practitioner

## Abstract

The goal of the present study was to analyze the prevalence and risk factors of suspected cancer diagnoses made by general practitioners (GP) in a population with subsequently confirmed cancer diagnoses in Germany. This study included patients aged 18 years or older who received an initial documentation of a confirmed cancer diagnosis from 1,262 German GP between January and December 2016 (index date). The main outcome measure of the study was the rate of suspected cancer diagnoses made by GP within one year prior to the index date. A multivariate logistic regression model was used to estimate the relationship between defined demographic and clinical variables and suspected cancer diagnoses. This study included 31,628 individuals. Within the year prior to the confirmed cancer diagnosis, 5% of the population received suspected cancer diagnoses. Patients in the age groups 41–50, 51–60, and 61–70 years were more likely to receive a suspected cancer diagnosis from a GP than those in the age group > 80 years (OR ranging from 1.30 to 1.38). Lung cancer, skin cancer, prostate cancer, and leukemia were associated with an increase in such odds when compared to cancers of the digestive organs (OR ranging from 1.56 to 2.26), whereas female genital organ cancers were associated with decreased odds (OR = 0.63). Overall, approximately 5% of patients received suspected diagnoses of cancer prior to their confirmed diagnoses. Suspected cancer diagnoses were associated with age and several types of cancer.

## INTRODUCTION

Cancer is one of the leading causes of morbidity and mortality in the world and accounted for around 8.8 million deaths in 2015 [1]. In Germany, the age-standardized incidence rate of cancer is approximately 324 cases per 100,000 men and 253 cases per 100,000 women [2]. Therefore, cancer has a major impact on health and economy in this country.

General practitioners (GP) play an important role in the diagnosis of cancer [3, 4]. A 2005 UK study including 65,192 patients showed that most individuals diagnosed with cancer saw their GP prior to being seen at a hospital [5]. The cancer diagnosis was even made by GP themselves in a small share of patients. Two years later, in 2007, Jones et al. found in that same country that the first occurrence of alarming symptoms (i.e. hematuria, hemoptysis, dysphagia, or rectal bleeding) was associated with an increased likelihood of cancer diagnosis by GP [6]. In 2016, a third UK analysis found that one of three patients visited a GP at least three times before being referred to a hospital physician and ultimately receiving a cancer diagnosis [7]. Interestingly, some types of cancer, such as pancreatic and thyroid cancers, were associated with a particularly high number of GP visits, suggesting that their recognition was more challenging compared to other cancers. Although these findings are of great interest, little is known about initial suspicions among GP concerning a malignant cause underlying the symptoms exhibited by their patients. Moreover, no German studies have yet been published on this topic.

Therefore, the goal of the present study was to analyze the prevalence of suspected cancer diagnoses made by GP in a population with subsequently confirmed cancer diagnoses in Germany.

## RESULTS

The baseline characteristics of patients are shown in Table 1. The present study included 31,628 individuals. The mean age was 67.4 years (SD: 15.0 years) and 52.2% of patients were women. The three most frequent cancer diagnoses were cancer of the digestive organs (16.5%), breast cancer (14.7%) and skin cancer (14.7%). Finally, 5% of the population received a suspected cancer diagnosis within the year prior to the confirmed cancer diagnosis. The results of the logistic regression models are displayed in Table 2. Patients in the age groups of 41–50, 51–60, and 61–70 years were more likely to receive a suspected cancer diagnosis from a GP than those in the age group >80 years (OR ranging from 1.30 to 1.38). Furthermore, people with more than four visits to a physician within one year prior to their index date had a higher chance of receiving a suspected cancer diagnosis than those with four visits or fewer (OR ranging from 2.57 to 3.17). Finally, lung cancer, skin cancer, prostate cancer, and leukemia were associated with an increase in such odds when compared to cancer of the digestive organs (OR ranging from 1.56 to 2.26), whereas female genital organ cancers were associated with decreased odds (OR = 0.63).

**Table 1:** Baseline characteristics of the population and prevalence of patients with suspected cancer diagnoses (QuintilesIMS, Disease Analyzer Database)

Variable	Patients with confirmed cancer diagnosis	Patients with suspected cancer diagnosis within one year prior to the date of confirmed cancer diagnosis	Patients with suspected cancer diagnosis (%)
N	31,628	1,590	5.0
**Demographic variables**			
Age (Mean, SD)	67.4 (15.0)	69.0 (13.7)	
≤ 40 years (N, %)	1,784 (5.6)	57	3.2
41–50 years (N, %)	2,482 (7.9)	101	4.1
51–60 years (N, %)	5,442 (17.2)	248	4.6
61–70 years (N, %)	6,815 (21.6)	377	5.5
71–80 years (N, %)	8,945 (28.3)	481	5.4
> 80 years (N, %)	6,160 (19.5)	326	5.3
Men (N, %)	15,126 (47.8)	849	5.6
Women (N, %)	16,502 (52.2)	741	4.5
Statutory health insurance coverage (*N*, %)	28,386 (89.8)	1,457	5.1
Private health insurance coverage (*N*, %)	3,242 (10.2)	133	4.1
**Number of visits to physician within one year prior to index date**			
≤ 4	20,524 (64.8)	536	2.6
5–8	4,449 (14.1)	368	8.3
9–12	2,872 (9.1)	277	9.6
> 12	3,783 (12.0)	409	10.8
Cancer diagnoses*			
Breast cancer	4,660 (14.7)	179	3.8
Female genital organ cancers	1,472 (4.7)	33	2.2
Prostate cancer	2,817 (8.9)	186	6.6
Lung cancer	2,202 (7.0)	175	8.0
Cancer of digestive organs	5,216 (16.5)	197	3.8
Urinary tract cancer	1,974 (6.2)	90	4.6
Skin cancer	4,656 (14.7)	428	9.2
Brain tumors	463 (1.5)	12	2.6
Lymphoma	1,246 (3.9)	49	3.9
Leukemia	1,375 (4.4)	73	5.3
Other cancer diagnoses	5,547 (17.5)	167	3.0
**Co-diagnoses**			
Diabetes	3,396 (10.7)	343	10.1
Coronary heart disease	2,124 (6.7)	217	10.2
Hypertension	8,108 (25.6)	746	9.2
Hyperlipidemia	3,519 (11.1)	336	9.6
Heart failure	1,282 (4.1)	128	10.0
Liver diseases	1,064 (3.4)	114	10.7
Diseases of esophagus, stomach and duodenum	3,406 (10.8)	303	8.9
Depression	1,821 (5.8)	162	8.9
Dementia	779 (2.5)	73	9.4

**Table 2:** Association between demographic/clinical variables and suspected cancer diagnoses in patients followed in general practitioner practices (logistic regression model)

Variables	Odds Ratio (95% CI)*	*p*-value*
**Demographic variables**		
Age ≤ 40 years versus > 80	1.26 (0.93–1.71)	0.131
Age 41–50 years versus > 80	1.38 (1.08–1.75)	0.010
Age 51–60 years versus > 80	1.31 (1.09–1.57)	0.004
Age 61–70 years versus > 80	1.30 (1.10–1.53)	0.002
Age 71–80 years versus > 80	1.07 (0.92–1.24)	0.411
men versus women	1.12 (0.99–1.26)	0.069
Private versus statutory health insurance coverage	0.86 (0.71–1.03)	0.109
**Number of visits to physician within one year prior to index date**		
5–8 versus ≤ 4	2.57 (2.22–2.98)	<0.001
9–12 versus ≤ 4	2.95 (2.50–3.48)	< 0.001
>12 versus ≤ 4	3.17 (2.69–3.74)	< 0.001
Cancer diagnoses*		
Lung cancer	2.26 (1.83–2.81)	< 0.001
Skin cancer	2.15 (1.80–2.57)	< 0.001
Prostate cancer	1.75 (1.41–2.18)	< 0.001
Leukemia	1.56 (1.18–2.06)	0.002
Breast cancer	1.22 (0.98–1.52)	0.077
Urinary tract cancer	1.14 (0.88–1.48)	0.314
Lymphomas	1.14 (0.82–1.57)	0.442
Brain tumors	0.86 (0.48–1.53)	0.612
Female genital organ cancers	0.63 (0.43–0.92)	0.017

*Reference group is cancer of digestive organs, the most frequent cancer diagnosis in general practitioner practices.

## DISCUSSION

The present study of 31,628 patients followed in German practices showed that 5% of the population received suspected cancer diagnoses from GP within the year prior to the confirmed cancer diagnoses. Younger individuals were more likely to receive such a diagnosis than older individuals. Compared with cancer of the digestive organs, four types of cancer were positively associated with a suspected cancer diagnosis: lung cancer, skin cancer, prostate cancer, and leukemia. In contrast, patients with female genital organ cancers were less likely to receive a suspected cancer diagnosis prior to a confirmed cancer diagnosis.

In recent years, several authors have underlined the key role played by GP in the diagnosis of cancer. In 2005, Allgar and Neal aimed to explore this role in the UK using data of 65,192 patients diagnosed with six different types of cancer (breast, colorectal, lung, ovarian, prostate, and non-Hodgkin's lymphoma (NHL)) [5]. The researchers in this study found that most individuals saw their GP prior to being seen by a hospital physician; this share increased from 72% in people with breast cancer to 90% in those with colorectal cancer. Higher age was negatively associated with the likelihood of seeing a GP prior to a hospital appointment in all cancers except ovarian cancer. Furthermore, cancer diagnoses were actually made by GP themselves in a small share of the population (8–19%). Compared to patients with breast cancer, those with colorectal, lung, ovarian, prostate, and NHL had a higher chance of receiving a diagnosis from their GP. Again, it was found that younger patients tended to more frequently receive a diagnosis directly from their GP compared to older patients.

Later, in 2007, Jones and colleagues aimed to evaluate the association between alarming symptoms and cancer diagnosis in a population of 762,325 patients aged 15 years or older and followed in 128 GP practices in the UK [6]. The authors found that the three-year positive predictive value of hematuria, hemoptysis, dysphagia, and rectal bleeding ranged from 2.0% to 7.5%. They further found that predictive value increased with age and reached 17.1% in men aged 75–84 years with hemoptysis. This work suggests that GP are often able to make an early diagnosis of cancer in older patients with alarming symptoms. Although the role of GP in the diagnosis of cancer is of great importance, a 2009 Scottish analysis showed that this role widely varies from one cancer to another [12]. The time from first symptoms to presentation to a GP ranged from two days in patients subsequently diagnosed with bladder cancer to 30 days in those subsequently diagnosed with head and neck cancer. For four different types of cancer (prostate, colorectal, melanoma, and head and neck cancers), 25% of patients visited their GP more than 2 months after their first symptoms. Furthermore, the priority with which GP referred patients to specialists widely varied between tumor groups, with 77.5% of breast cancer patients and 44.7% of those with prostate cancer classified as needing an urgent referral to secondary care, respectively.

More recently, in 2014, Jensen et al. conducted a population-based, cross-sectional study of incident cancer patients in Denmark who visited their GP prior to being diagnosed with cancer [13]. They used data from a national register and from GP questionnaires, in which the GPs were asked to classify the patient's symptoms into “vague”, “serious,” or “alarming”. Authors found that cancer diagnosis involved a GP in almost three out of four patients (73.5%). In approximately 48% of these individuals, GP considered the symptoms as alarming and identified cancer as the most likely diagnosis. For around 37% of the population, GP activated a standardized cancer patient procedure in order to ensure fast diagnosis. It was estimated that individuals displaying symptoms classified as “vague” were less likely to benefit from this fast diagnosis track than those with alarming symptoms. Finally, the median delay from first presentation of symptoms in primary care to diagnosis was 34 days longer in people with “vague” symptoms compared to those with alarming symptoms.

Finally, in 2016, Lacey and colleagues aimed to assess the variations in the number of GP visits preceding cancer diagnosis, as well as in the delay between first symptoms and referral to a hospital physician [7]. This Australian study of 1,248 patients found that around one out of three patients had been seen by a GP at least three times prior to referral to a hospital specialist. Individuals with pancreatic, thyroid, and vulvar cancer and those with multiple myeloma were more likely to have at least three GP visits than those with rectal cancer, whereas people with breast, cervical, and endometrial cancer and people with melanoma exhibited a lower risk for such a high number of visits. The type of tumor also had an impact on the delay between first symptoms and referral to a hospital physician, the delay being particularly high in patients subsequently diagnosed with prostate and colon cancer. These latter findings suggest that cancer diagnoses made by GP are associated with various levels of difficulty, with the levels depending on the type of cancer.

In line with these previous works, we found in the present German study that GP suspected cancer in a certain proportion (5%) of a population that subsequently received confirmed diagnoses of cancer. Another important finding of this retrospective analysis is that the risk of receiving a suspected cancer diagnosis significantly varied with the type of tumor. Patients with lung, skin, or prostate cancer and those with leukemia had a higher chance of receiving such a diagnosis when compared with patients subsequently diagnosed with cancer of the digestive organs. This result might be explained by the fact that these different cancers are often associated with alarming symptoms or signs and with abnormal routine tests: hemoptysis for lung cancer [14, 15], precancerous lesions for skin cancer [16, 17], abnormal digital rectal examination for prostate cancer [18], and chronic fever for leukemia [19, 20]. In contrast, people affected by cancer of the digestive organs are often asymptomatic and are diagnosed at an advanced stage of the disease [21]. In the case of female genital organ cancers, one has to consider that diagnosis, treatment, and management of women with genital symptoms are mostly performed in gynecological practices. Therefore, GP may directly refer symptomatic women to a gynecologist without making an initial diagnosis. We further found that higher age was negatively associated with the likelihood of receiving a suspected cancer diagnosis. This result is in line with the literature, as Allgar and Neal previously showed that younger people were more likely to receive a cancer diagnosis from their GP compared with older people [5]. Our hypothesis is that older individuals are often affected by chronic disorders with chronic symptoms [22–24], indirectly leading GP to underestimate the seriousness of the symptoms presented by these patients. Finally, we found that the number of consultations was associated with an increase in the chance of receiving a suspected cancer diagnosis. Therefore, GP are more likely to suspect cancer when symptoms are present for a certain amount of time and are not alleviated by symptomatic treatments. This study also shows that time is important for physicians so that they can develop a better understanding of their patients’ potential diseases.

This study was subject to several limitations. In general, retrospective primary care database analyses are limited by the validity and completeness of the data on which they are based. The estimated share of 5% is relatively small. This study includes different cancer diagnoses and gynecologists rather than GPs are responsible for diagnosing breast and female genital organ cancers. The main limitation is that suspected and confirmed cancer diagnoses relied solely on ICD codes entered by primary care physicians. A full list of diagnoses that were used by GPs to document the suspected cancer diagnoses was not available. It is possible that only symptoms (for example, bleeding or pain) and not cancer diagnoses are documented in the event of suspected cancer, so as not to trouble the patients. Furthermore, the database did not include any valid information pertaining to TNM classification. Data on socioeconomic status and lifestyle-related risk factors were also unavailable. Finally, information on the delay between first symptoms and presentation to a GP was lacking. The main strengths of this study are the number of patients and the types of cancer included in the analysis.

Overall, approximately 5% of cancer patients received a suspected diagnosis of cancer by a GP prior to a confirmed diagnosis. Suspected cancer diagnosis was associated with age and several types of cancer. Additional studies are needed to gain a better understanding of the role played by GP in the management of individuals potentially affected by cancer.

## MATERIALS AND METHODS

### Database

This retrospective study is based on data from the Disease Analyzer database (QuintilesIMS), which compiles demographic, clinical, and pharmaceutical data obtained in an anonymous format from computer systems used in clinical practices [8]. The quality and exactness of the data (e.g., diagnoses and drug prescriptions) are regularly assessed by QuintilesIMS. Using prescription statistics for several drugs and age groups for several diagnoses, the Disease Analyzer database was found to be a representative database of clinical practices in Germany [8]. Finally, several studies focusing on cancer and using the same database have already been published [9–11].

### Study population

This study included patients aged 18 years or older who received an initial documentation of a confirmed cancer diagnosis (ICD 10: C00-C96) from 1,262 German GP between January and December 2016 (index date).

### Study outcome and independent variables

The main outcome measure of the study was the rate of suspected cancer diagnoses made by GP within one year prior to the index date. Patients with a suspected cancer diagnosis were further sent to specialists in order to exclude such diagnosis. Demographic data included age groups (≤ 40, 41–50, 51–60, 61–70, 71–80, and > 80 years), gender, health insurance coverage (private vs. statutory), and frequency of visits to physician within one year prior to the index date. Clinical data included different types of cancer (breast cancer (C50), female genital organ cancer (C51–58), prostate cancer (C61), lung cancer (C34), cancer of digestive organs (C15-C26), urinary tract cancer (C64–68), skin cancer (C43–44), brain tumors (C70, C71), lymphomas (C81–88), multiple myeloma and leukemia (C90–98), and all other cancer diagnoses included in a single group), diabetes (E10–14), coronary heart disease (I24, I25), hypertension (I10), hyperlipidemia (E78), heart failure (I50), liver diseases (K70–77), diseases of esophagus, stomach, and duodenum (K20–31), depression (F32, F33), and dementia (F01-F03, G30).

### Statistical analyses

A multivariate logistic regression model was used to estimate the relationship between defined demographic and clinical variables and suspected cancer diagnoses. A *p*-value < 0.05 was considered statistically significant. All analyses were carried out using SAS 9.3 (SAS Institute, Cary, USA).
